# Supplementary material for: Impact of Anti PD-1 Immunotherapy on HIV Reservoir and Anti-Viral Immune Responses in People Living with HIV and Cancer
Source: Cells. 2022 Mar 17;11(6):1015. doi: 10.3390/cells11061015 (PMC8946896; doi:10.3390/cells11061015)
Supplement: Supplementary file 1 [file cells-11-01015-s001.zip › cells-1602303-supplementary.pdf]

SUPPLEMENTARY MATERIAL

**Section**	**Content**	**Page**
Section I	Supplementary Methods	1
Section II	Supplementary Tables	4
Section III	Supplementary Figures	5
Section IV	References	19

SECTION I: Supplementary Methods

Viral stimulations before ICS assays are detailed below. All stimulations, except HBV, have been previously described.

- HIV-Gag pooled peptides 15mers overlapping by 11 amino acids and spanning the complete sequence of p17 and p24 (final concentration 2µg/mL of each peptide, provided by NeoSystem).[1].
- HIV-Gag pooled and RT and Nef pooled peptides 15mers overlapping by 11 amino acids and spanning the complete sequence of P1, P2, P6, P7, RT and Nef (final concentration 2µg/mL of each peptide, provided by NeoSystem) [1].
- EBV peptides 8-11mers representing immunodominant epitopes restricted to different class-I MHC molecules from EBNA-1, EBNA-2, EBNA-3A, EBNA-3B, EBNA-3C, LMP-2, BMLF-1, BZLF-1, - (final concentration 2µg/mL, provided by GeneCust Europe) [2].
- IE1 CMV 15mers peptides overlapping by 10 amino acids and spanning the complete sequence of IE1 (final concentration 2 µg/ml, provided by the « INSERM « Action-thématique-Concertée on antiviral immunity ») [3].
- PP65 CMV 15mers peptides overlapping by 10 amino acids and spanning the complete sequence of PP65 (final concentration 2 µg/ml, provided by the « INSERM « Action-thématique-Concertée on antiviral immunity »)[3].
- HBV 8-15mers peptides overlapping by 11 and spanning the complete sequence of Enveloppe (genotype A: one of the most common french genotype) and HBV peptides representing immunodominant epitopes restricted to different class-I MHC molecules from Enveloppe, Core, HbX and Polymerase (genotypes A and D: the 2 most common french genotype) (final concentration 2µg/mL, provided by Genecust, France) These ninety peptides were selected from immunodominant peptides, prioritizing peptides

from HBV genotype A and D because of their frequency in Europe and prioritizing the more frequent european HLAs, and from the complete sequence of Enveloppe protein with overlapping peptides (genotype A) [4–8]. HbYX peptides represent peptides derived from HbX protein with tyrosine substitutions at position 1 and whose immunogenicity was demonstrated. HBV peptides sequences, genotype, HLA restriction and amino acids number are summarized in **Supplementary Table 1**.

- HCV 18mers peptides overlapping by 11 amino acids and spanning the complete sequence of NS-3, NS-5a, NS-5b and core (final concentration 0,5 µg/mL, provided by the NIH-AIDS-Reagent-Program) [9].
- HHV8 9-22mers peptides representing immunodominant epitopes from lytic viral glycoprotein (gp) B246, gp H, gp 35, and latent viral LANA-1, K12, K15,ORF-57 (final concentration 2 µg/mL, provided by Epitop) [10].

SECTION II: Supplementary Table

Table S1: **Immunodominant HBV peptides sequences, genotype, HLA restriction and amino acids number.**

HBV peptides	Sequence	HBV genotype	HLA restriction	Amino acids number
Core 18-27	FLPSDFFPSV	A and D	A2	10
Core 107-115	CLTFGRETV	A and D	A2	9
Env 183-191	FLLTRILTI	A and D	A2	9
Env 335-343	WLSLLVPFV	A and D	A2	9
Env 338-347	LLVPFVQWFV	A and D	A2	10
Env 348-357	GLSPTVWLSA	A	A2	10
Env 348-357	GLSPTVWLSV	D	A2	10
Pol 455-463	GLSRYVARL	A and D	A2	9
Pol 502-510	KLHLYSHPI	A and D	A2	9
Pol 575-583	FLLSLGIHLFLLSLGIHL	A and D	A2	19
Pol 655-663	ALMPLYACI	A and D	A2	9
Pol 816-824	SLYAVSPSV	A	A2	9
Pol 816-824	SLYADSPSV	D	A2	9
Core 419-428	DLLDTASALY	A and D	A2	10
Pol 538-546	YMDDVVLGA	A and D	A2	9
Pol 149-159	HTLWKAGILYK	A and D	A11	11
Pol 388-397	LVVDFSQFSR	A and D	A11	10
Pol 531-539	SAICSVVRR	A and D	A11	9
Core 141-151	STLPETTVVRR	A and D	A11	11
HbX1 4-12	RLCCQLDPA	A and D	A2	9
HbX6 84-92	NAHQILPKV	A	A2	9
HbX6 84-92	NAHQFLPKV	D	A2	9
HbYX7 92-100	YLHKRTLGL	A and D	A2	9
HbYX10 115-123	YLFKDWEEL	A and D	A2	9
HbYX12 99-108	YLSAMSTTDL	A	A2	10
HbYX12 99-108	YLSVMSTTDL	D	A2	10
Core 8-16	EFGATVELL	A and D	A2	9
Core 75-83	LEDPASRDL	A	B4001	9
Core 75-83	LEDPASREL	D	B4001	9
Core 88-96	YVNTNMGLK	A and D	A1101	9
Core 117-125	EYLVQFGVW	A and D	A2402	9
Core 141-151	TLPETTVVRR	A and D	A1101	10
Env 61-70	GLLGWSPQA	A and D	A0201	9
Env 171-180	FLGPLLVLQA	A and D	C0801	10
Env 356-364	SAIWMMWYW	A and D	B5801	9
Env 370-379	SIVSPFIPLL	A	A0201	10
Env 370-379	SILSPFLPLL	D	A0201	10
Pol 165-174	ASFCGSPYSW	A and D	B5801	10
Pol 386-395	SRLVVDFSQF	A and D	B1301	10
Pol 756-764	KYTSFPWLL	A and D	A2402	10
Env 177-185	VLQAGFFIL	A and D	A2	9
Env 204-212	FLGGTRVCL	A	A2	9
Env 204-212	FLGGTTVCL	D	A2	9
Env 250-258	LLCLIFLLV	A and D	A2	9
Env 260-269	LLDYQGMLPV	A and D	A2	10

Abbreviation: Env : enveloppe : Pol : polymerase.

SECTION III: Supplementary Figures

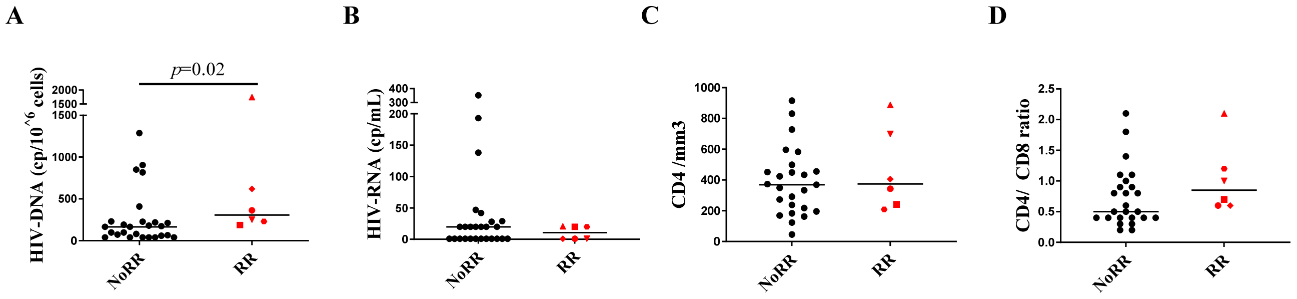

Figure S1. Cell-associated HIV DNA at baseline is higher in patients with virological reduction compared to the others. At baseline **A**, HIV-DNA (cp/$10^{\wedge 6}$ cells). **B**, HIV-RNA (cp/mL). **C**, CD4 count /mm3. **D**, CD4/ CD8 ratio. NoRR: patients without reservoir reduction; RR: patients with reservoir reduction. Mann-Whitney test. For the HIV-RNA results, the undetectable results were depicted at the threshold of detection (1 cp/mL), the detectable but unmeasurable results (1-<20 cp/mL) were depicted at the threshold detection (20 cps/mL). Experiments were monoplicates.

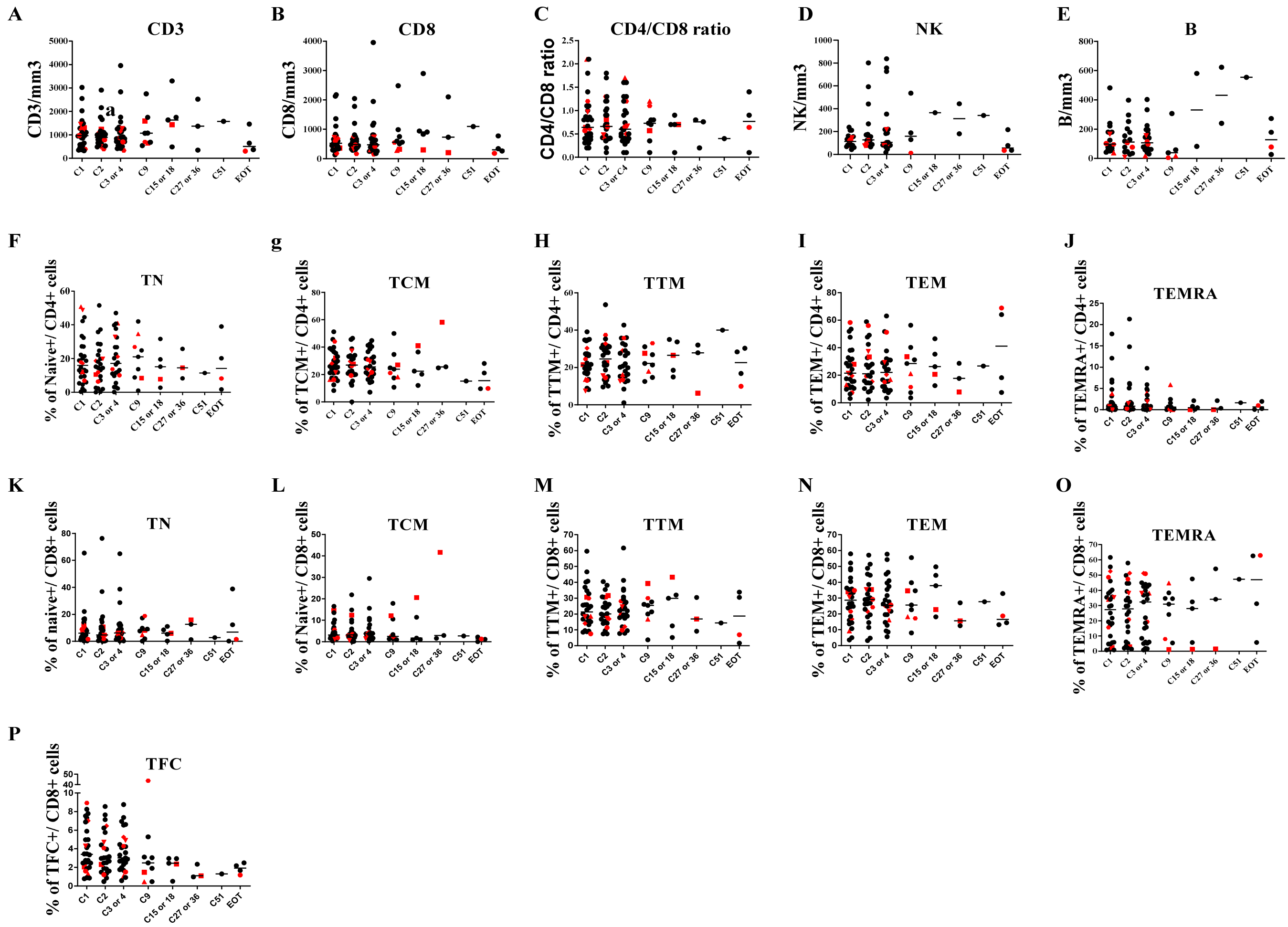

Figure S2. Lymphocytes' subsets and subpopulations are not modified under anti-PD-1 therapy. **A**, CD3 count (/mm3). **B**, CD8 count (/mm3). **C**, CD4/ CD8 ratio. **D**, NK count (/mm3). **E**, B count (/mm3) % of **F**, naive CD4+ T cells. **G**, central memory CD4+ T cells. **H**. transitional memory CD4+ T cells. **I**. effector memory CD4+ T cells. **J**, TEMRA CD4+ T cells. **K**, naive CD8+ T cells. **L**, central memory CD8+ T cells. **M**. transitional memory CD8+ T cells. **N**. effector memory CD8+ T cells. **O**, TEMRA CD8+ T cells. **P**, follicular cytotoxic CD8+ T cells. Abbreviations. TN: naïve T cells; TCM: central memory T cells; TTM: transitional memory T cells, TEM: effector memory T cells; TEMRA: effector memory RA T cells; TFC: follicular cytotoxic T cells. EOT, end of treatment. Red symbols denote the RR patients. Experiments were monoplicates.

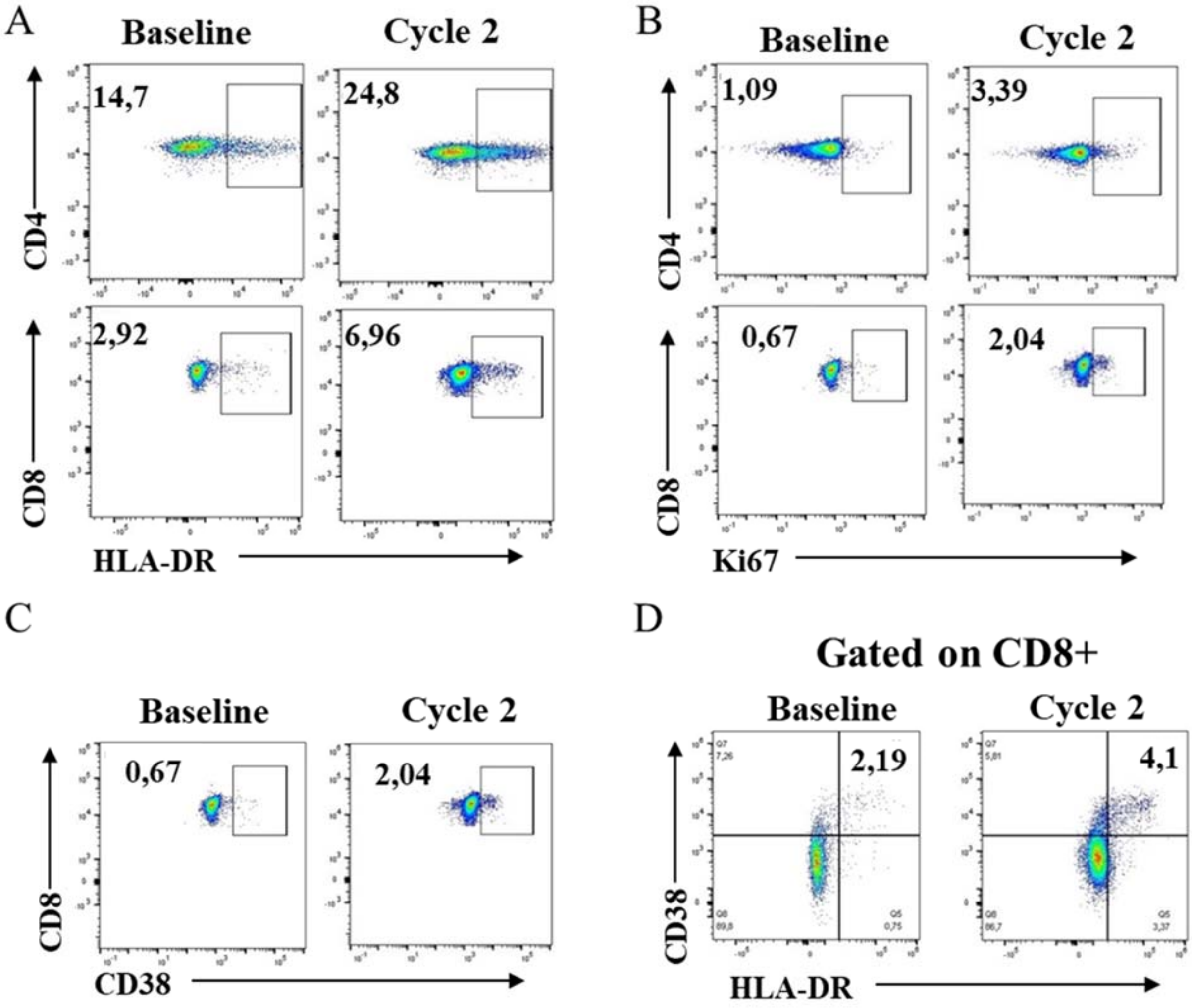

Figure S3. Example dot plots of **A**, HLA-DR and of **B**, Ki-67 expression among CD4 and CD8 T cells at baseline and at cycle 2; of **C**, CD38 and of **D**, CD38+ HLA-DR+ expression among CD8 T cells at baseline and at cycle 2. Experiments were monoplicates.

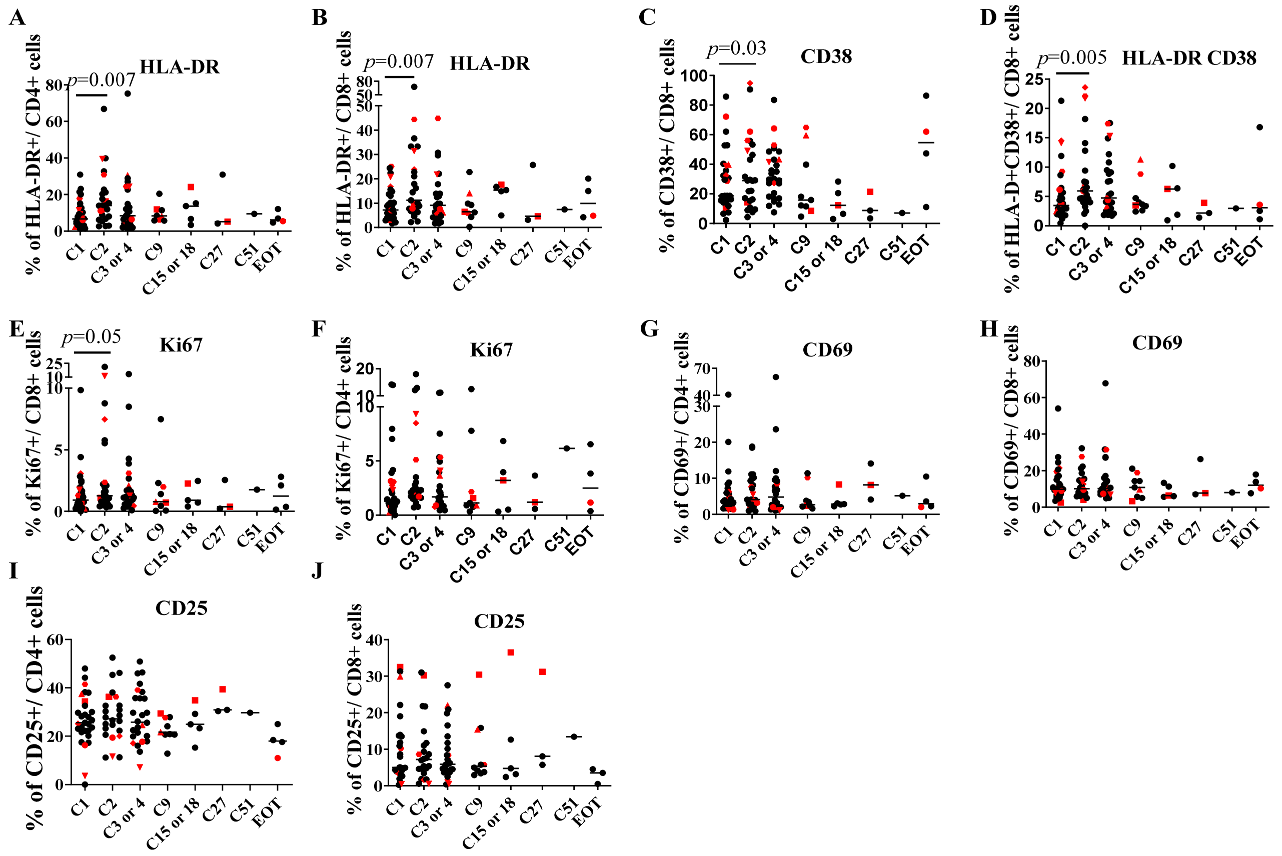

Figure S4. CD69 and CD25 expression on CD4+ and CD8+ T cells are not modified under anti-PD-1 therapy. % of **A** HLA-DR+ CD4+ T cells. **B**, HLA-DR+ CD8+ T cells. **C**, CD38+ CD8+ T cells. **D**, CD38+ HLA-DR+ CD8+ T cells. **E**, Ki67+ CD4+ T cells. **F**, Ki67+ CD8+ T cells. **G**, CD69+ CD4+ T cells. **H**, CD69+ CD8+ T cells. **I**, CD25+ CD4+ T cells. **J,** CD25+ CD8+ T cells. EOT, end of treatment. Red symbols denote the six RR patients. Bonferroni corrected p-value: $p \leq 0.007$, Wilcoxon-matched pairs signed Rank test. Experiments were monoplicates.

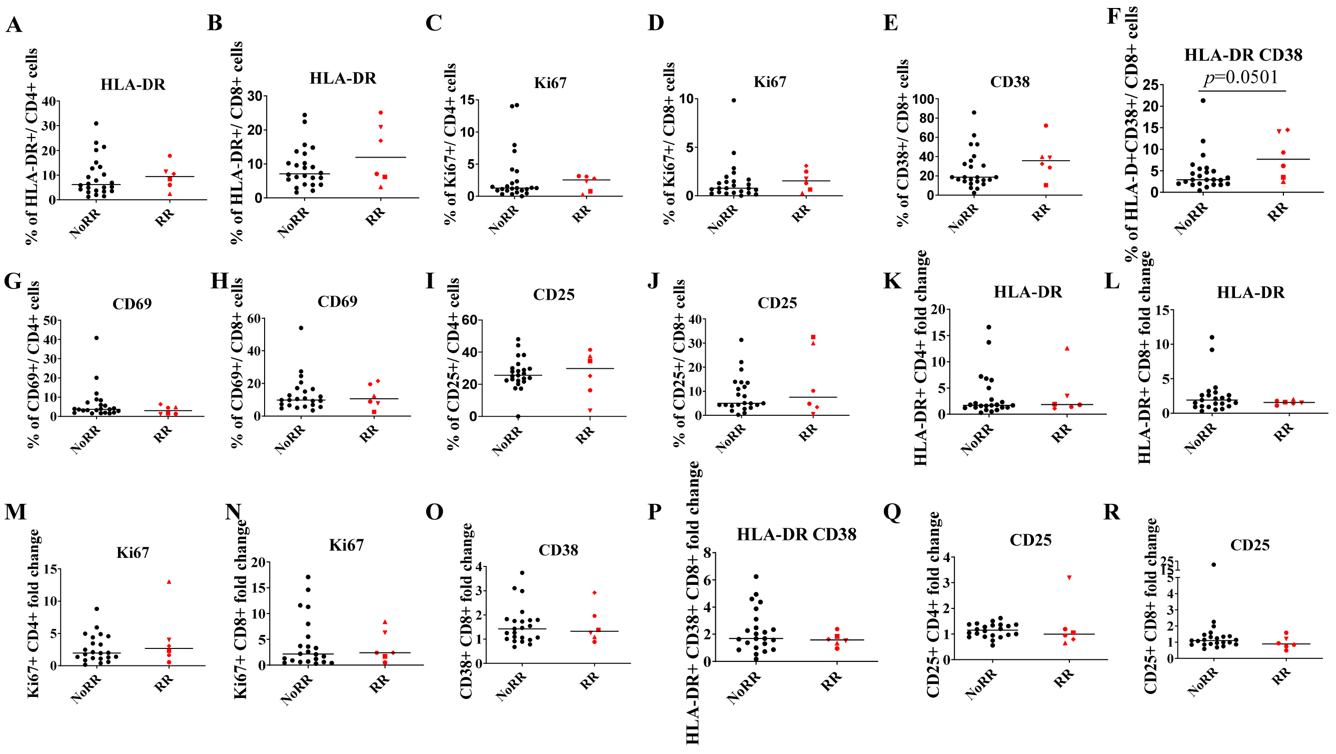

Figure S5. Activation markers expression at baseline and activation markers kinetics are not different between the RR patients and the others. % at baseline of **A**, HLA-DR+ CD4+ T cells. **B**, HLA-DR+ CD8+ T cells. **C**, Ki67+ CD4+ T cells. **D**, Ki67+ CD8+ T cells. **E**, CD38+ CD8+ T cells. **F**, CD38+ HLA-DR+ CD8+ T cells. **G**, CD69+ CD4+ T cells. **H**, CD69+ CD8+ T cells. **I**, CD25+ CD4+ T cells. **J**, CD25+ CD8+ T cells. Highest fold change at C2 or C3 of **J**, HLA-DR+ CD4+ T cells. **K**, HLA-DR+ CD8+ T cells. **L**, Ki67+ CD4+ T cells. **M**, Ki67+ CD8+ T cells. **N**, CD38+ CD8+ T cells. **O**, CD38+ HLA-DR+ CD8+ T cells. **P** CD25+ CD4+ T cells. **Q**, CD25+ CD8+ T cells. RR: patients with reservoir reduction, NoRR: patients without reservoir reduction. Mann-Whitney test. Experiments were monoplicates.

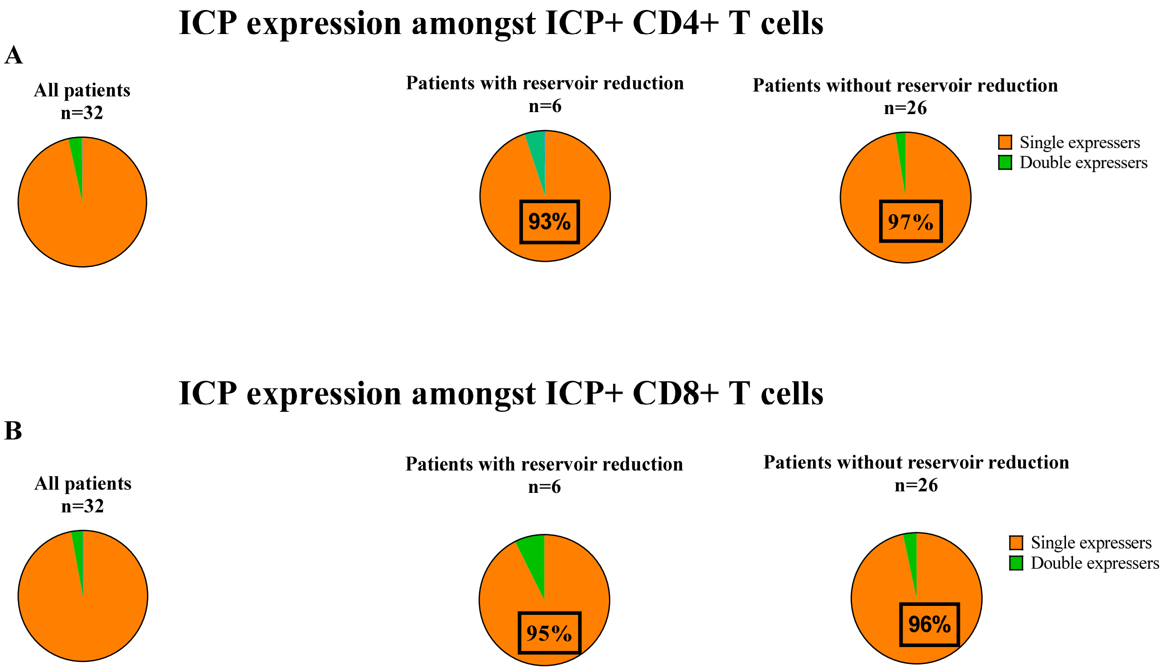

Figure S6. PD-1, CTLA-4 and TIM-3 expression profile on total CD8+ and CD4+ T cells at baseline before anti-PD-1 therapy. The percentages of (**A**) CD4+ or (**B**) CD8+ among CD4+ICP+ or CD8+ ICP+ T cells expressing one (single), two (double) or three (triple) ICP among PD-1, CTLA-4 and TIM-3 are represented for all patients at the left and for the RR patients (n=6) and the others (n=26) at the right. Experiments were monoplicates.

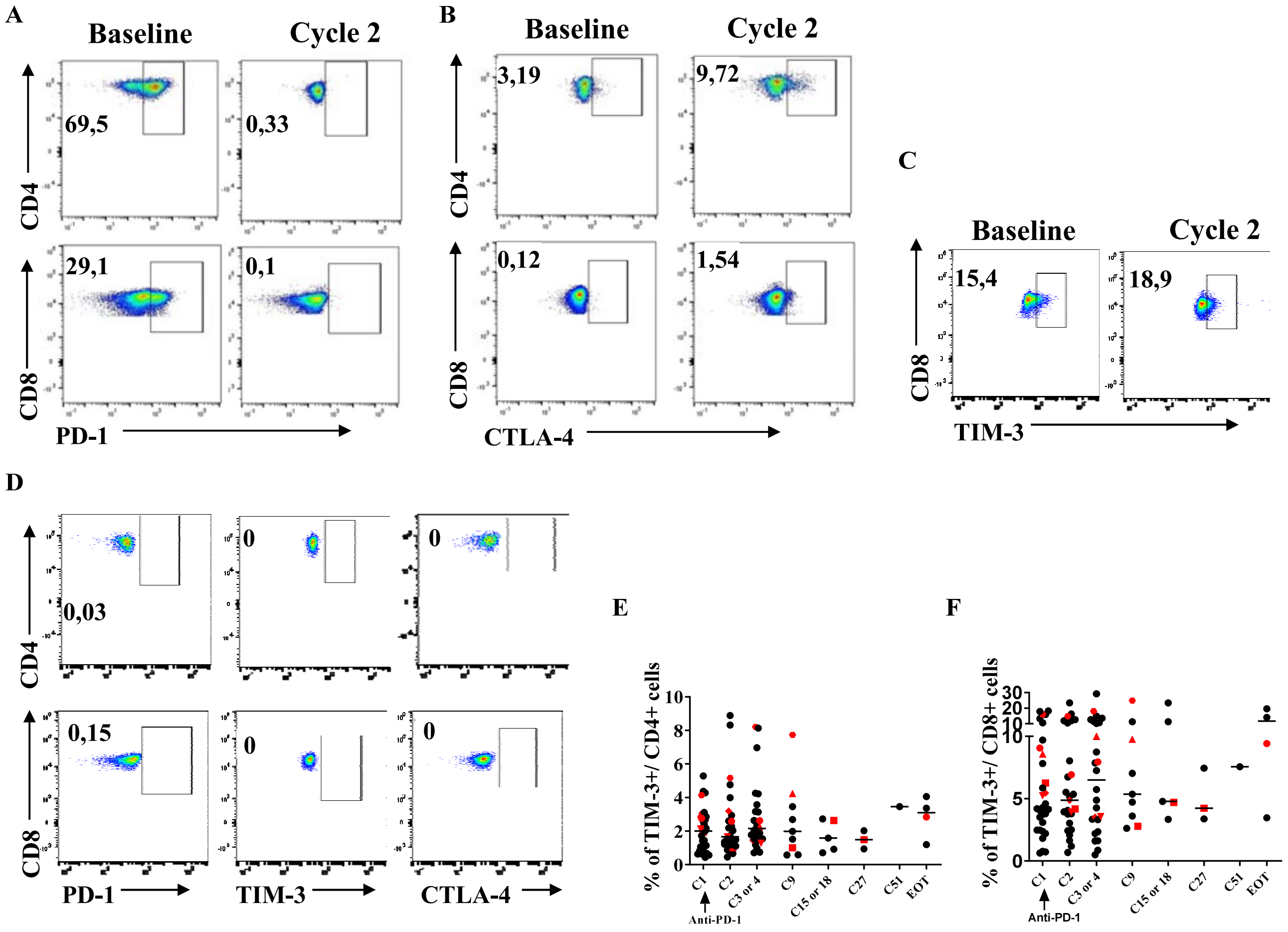

Figure S7. **Example dot plots** of **A**, PD-1, **B**, CTLA-4 and **C**, TIM-3 expression among CD4+ and CD8+ T cells at baseline and at cycle 2. **D**, **Fluorescence Minus One controls** of PD-1, CTLA-4 and TIM-3. **TIM-3 expression** in **E**, CD4+ and in **F**, CD8+ T cells. Experiments were monoplicates.

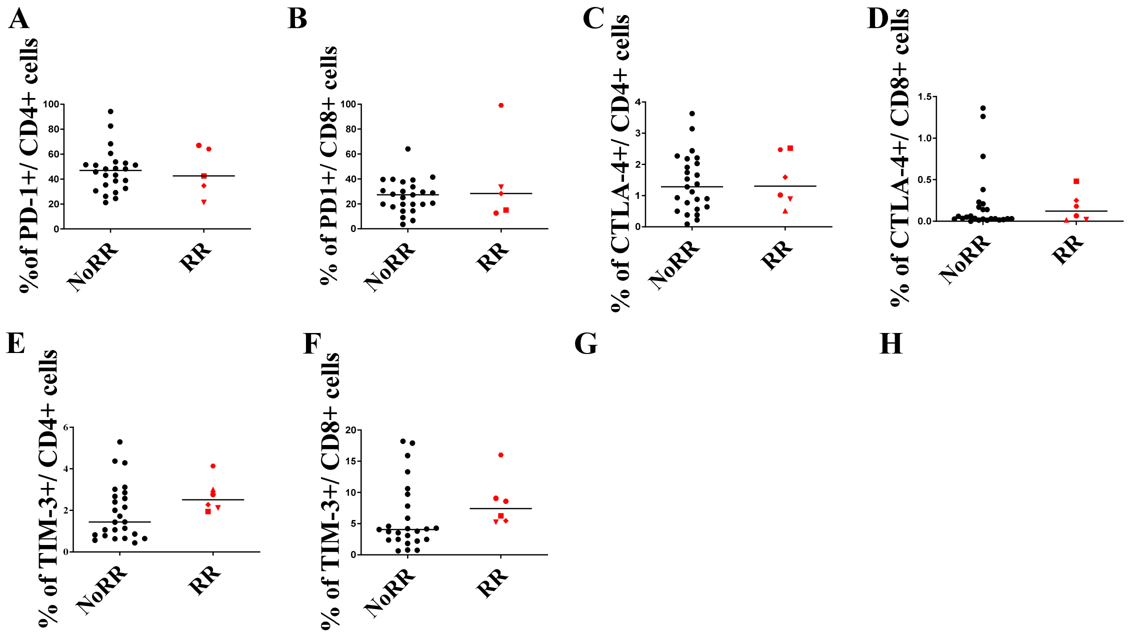

Figure S8. ICP expression at baseline are not different between the RR patients and the others. % at baseline of **A**, PD-1+ CD4+ T cells. **B**, PD-1+ CD8+ T cells. **C,** CTLA-4+ CD4+ T cells. **D,** CTLA-4+ CD8+ T cells. **E**, TIM-3+ CD4+ T cells. **F**, TIM-3+ CD8+ T cells. Highest fold change at C2 or C3 of **G**, CTLA-4+ CD4+ T cells. **H,** TIM-3+ CD4+ T cells. RR: patients with reservoir reduction, NoRR: patients without reservoir reduction. Experiments were monoplicates.

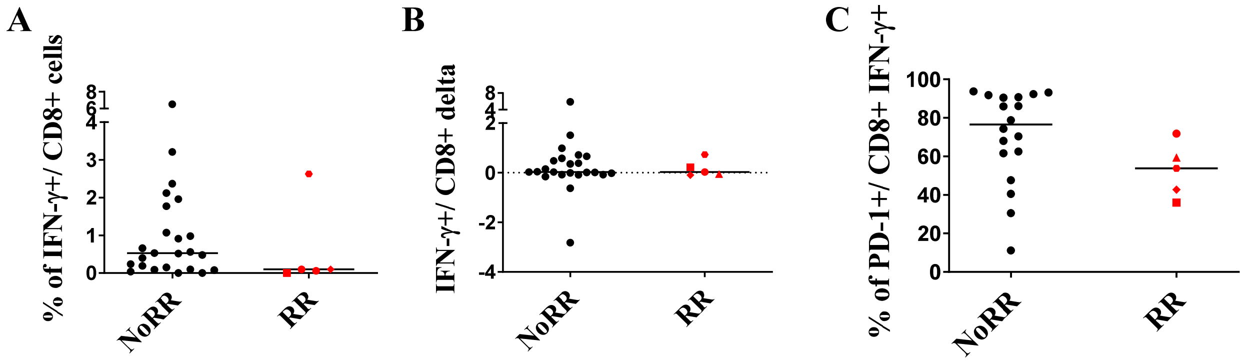

Figure S9. HIV-specific CD8+ T cells proportions at baseline and kinetics are not different between the RR patients and the others. HIV-specific CD8 + T cells are defines as IFN- ɣ producing CD8 + T cells. **A,** % of HIV-specific CD8+ T cells at baseline. **B**, HIV-specific CD8+ T cells best delta at C2 or C3. **C**, % of PD-1 on HIV-specific CD8+ T cells at baseline. RR: patients with reservoir reduction, NoRR: patients without reservoir reduction. Experiments were monoplicates.

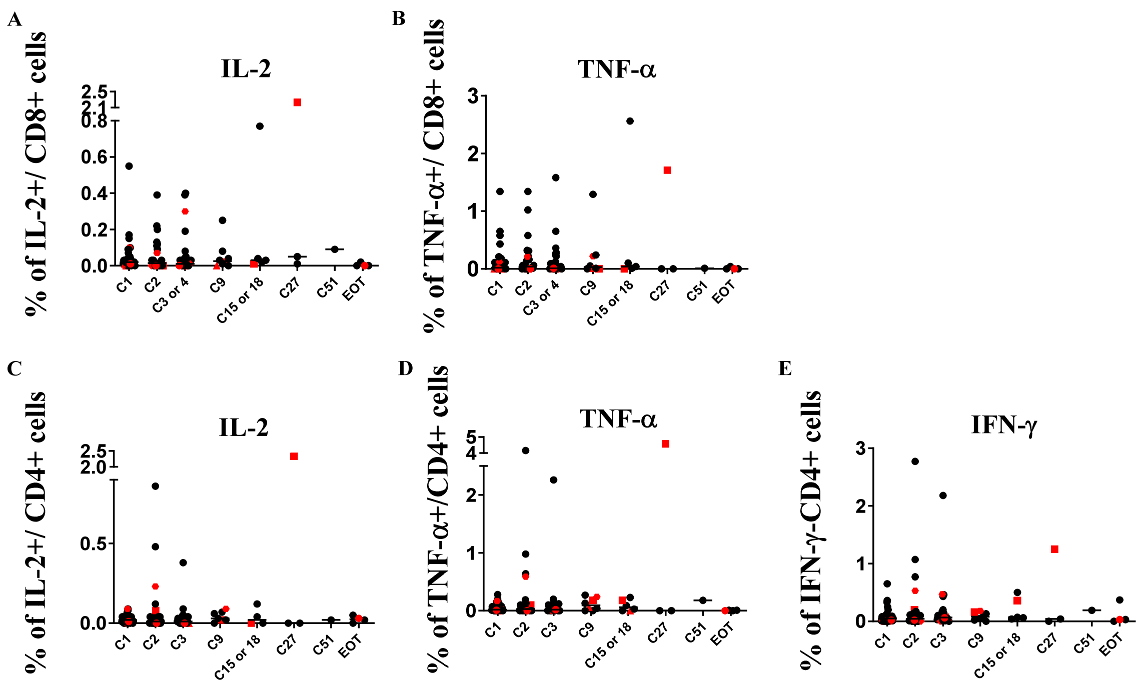

Figure S10. Intra-cellular cytokine expression after HIV-peptide stimulation is not modified under anti-PD-1 therapy. Thawed PBMC were stimulated during 6 hours with HIV peptides (15mers overlapping peptides covering RT, Nef and Gag) and incubated overnight. Then, they were stained with intra-cellular cytokine (IFN- ɣ, IL-2, TNF-α). % of **A**, IL-2+ CD8+ T cells. **B**, TNF-α+ CD8+ T cells. **C**, IL-2+ CD4+ T cells. **D**, TNF-α+ CD4+ T cells. **E**, IFN- ɣ+ CD4+ T cells. Red symbols denote the 6 patients with reservoir reduction. Experiments were monoplicates.

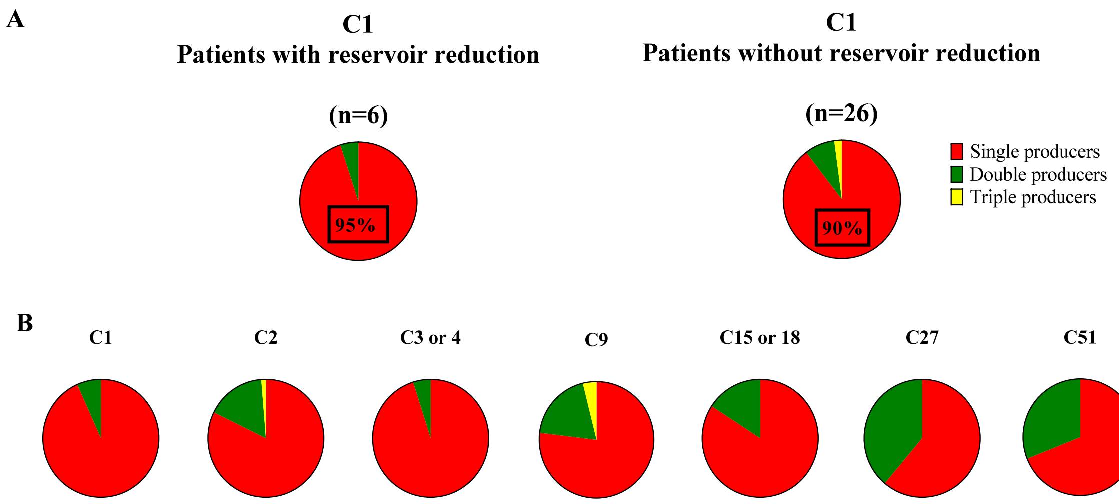

Figure S11. Polyfunctionality profile amongst HIV-specific CD8+ T cells is not modified under anti-PD-1 therapy. Cytokine production (IFN-ɣ, IL-2, TNF-α) was assessed with intracellular cytokine staining after stimulation during 6 hours with HIV-15mers overlapping peptides. **A**, Example dot plots of TNF-α + IFN- ɣ + CD8+ T cells. **B**, cytokine production profile at baseline in patients RR and NoRR. **C**, cytokine production profile for all patients overtime. Red pies represent the percentage of CD8+ T cells producing only 1 cytokine, green pies the percentage of CD8+ T cells producing 2 cytokines and yellow pies the percentage of CD8+ T cells producing 3 cytokines. Experiments were monoplicates.

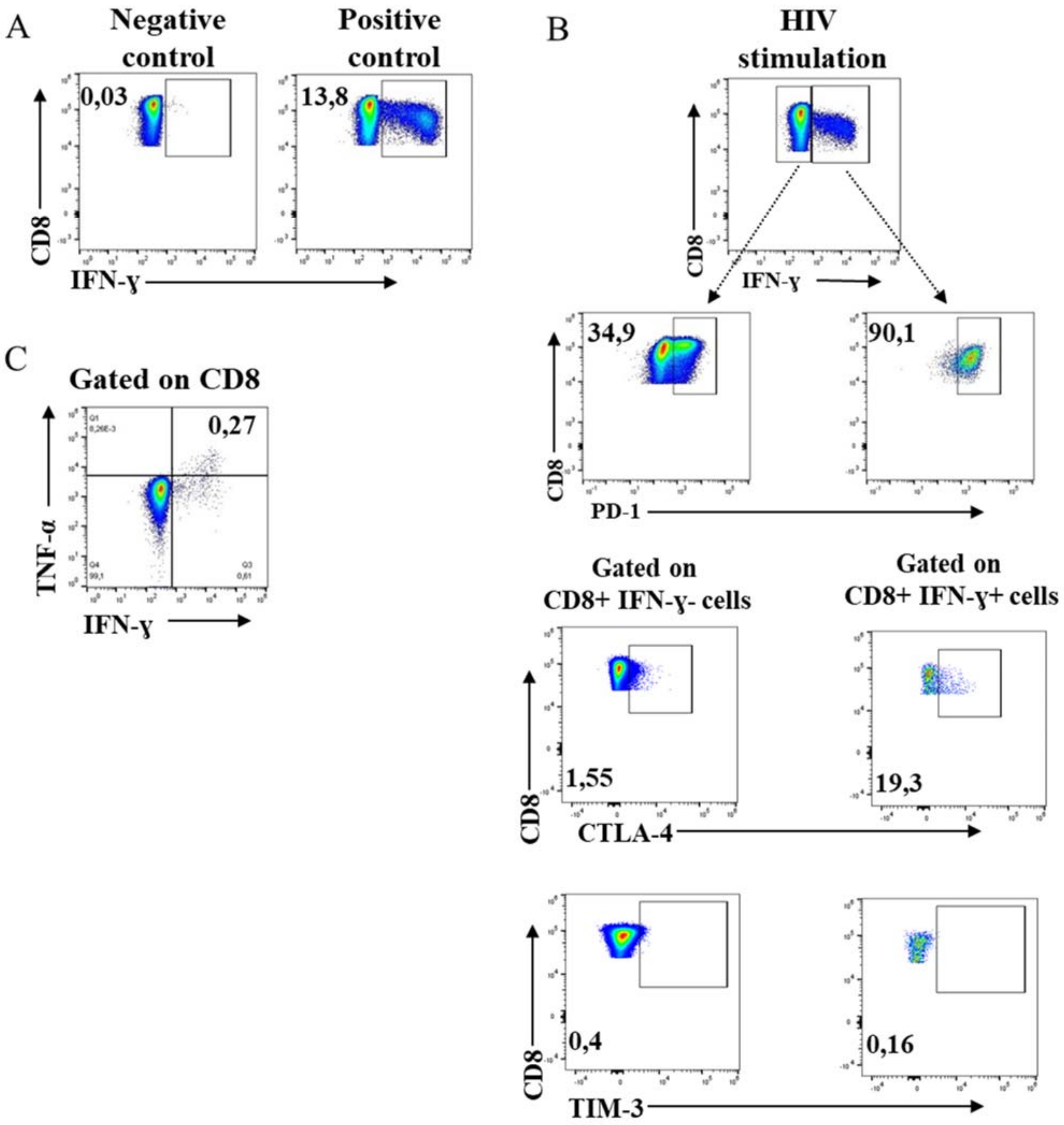

Figure S12. **Example dot plots** of **A**, IFN-ɣ+ CD8+ T cells after stimulation with medium alone (negative control on the left) and with staphylococcal enterotoxin B toxin (positive control on the right). PD-1, of **B**, PD-1, CTLA-4 and TIM-3 expression among HIV non-specific (IFN-ɣ-) and HIV-specific (IFN-ɣ+) CD8+ T cells after HIV stimulation at baseline, of, **C**, CD8+ cells producing both IFN-ɣ and TNF-α. Experiments were monoplicates.

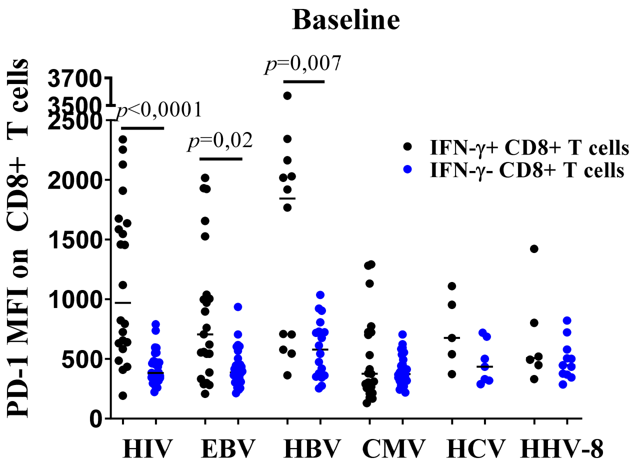

Figure S13. High levels of mean fluorescence intensity (MFI) are detected on HIV, EBV and HBV-specific CD8+ T cells baseline after viral stimulation. PD-1 MFI among specific (black spots) and non-specific (blue square) CD8+ T cells at baseline depending on viral (HIV, EBV, HBV, CMV, HCV and HHV-8) stimulations during 6 hours. Mann Whitney test. Experiments were monoplicates.

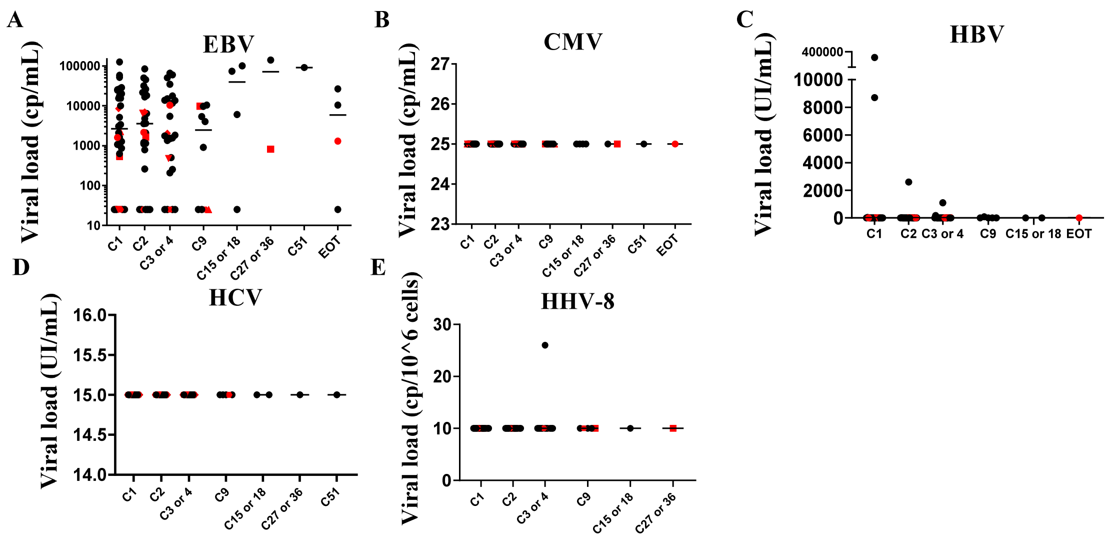

Figure S14. **Viral loads under ICB**. **A**. EBV viral load (cp/mL) **B.** CMV viral load (cp/mL). **C**, HBV viral load (UI/mL). **D**, HCV viral load (UI/mL). **E**, HHV8 viral load (cp/10^6 cells). Viral loads were assessed overtime for patients with positive serology at baseline. Red symbols denote the six patients with reservoir reduction. Undetectable results were depicted at the threshold of detection. Experiments were monoplicates.

SECTION IV: Supplementary References

[1] Samri A, Durier C, Urrutia A, Sanchez I, Gahery-Segard H, Imbart S, et al. Evaluation of the interlaboratory concordance in quantification of human immunodeficiency virus-specific T cells with a gamma interferon enzyme-linked immunospot assay. Clin Vaccine Immunol CVI 2006;13:684–97. https://doi.org/10.1128/CVI.00387-05.

[2] Nakid-Cordero C, Arzouk N, Gauthier N, Tarantino N, Larsen M, Choquet S, et al. Skewed T cell responses to Epstein-Barr virus in long-term asymptomatic kidney transplant recipients. PloS One 2019;14:e0224211. https://doi.org/10.1371/journal.pone.0224211.

[3] Sacre K, Carcelain G, Cassoux N, Fillet A-M, Costagliola D, Vittecoq D, et al. Repertoire, diversity, and differentiation of specific CD8 T cells are associated with immune protection against human cytomegalovirus disease. J Exp Med 2005;201:1999–2010. https://doi.org/10.1084/jem.20042408.

[4] Webster G, Bertoletti A. Quantity and quality of virus-specific CD8 cell response: relevance to the design of a therapeutic vaccine for chronic HBV infection. Mol Immunol 2001;38:467–73. https://doi.org/10.1016/S0161-5890(01)00082-7.

[5] Webster GJM, Reignat S, Brown D, Ogg GS, Jones L, Seneviratne SL, et al. Longitudinal analysis of CD8+ T cells specific for structural and nonstructural hepatitis B virus proteins in patients with chronic hepatitis B: implications for immunotherapy. J Virol 2004;78:5707–19. https://doi.org/10.1128/JVI.78.11.5707-5719.2004.

[6] Depla E, Van der Aa A, Livingston BD, Crimi C, Allosery K, De Brabandere V, et al. Rational Design of a Multiepitope Vaccine Encoding T-Lymphocyte Epitopes for Treatment of Chronic Hepatitis B Virus Infections. J Virol 2008;82:435–50. https://doi.org/10.1128/JVI.01505-07.

[7] Malmassari S, Lone YC, Zhang M, Transy C, Michel M-L. In vivo hierarchy of immunodominant and subdominant HLA-A*0201-restricted T-cell epitopes of HBx antigen of hepatitis B virus. Microbes Infect 2005;7:626–34. https://doi.org/10.1016/j.micinf.2004.12.022.

[8] Tsai S-L, Lee T-H, Chien R-N, Liao S-K, Lin C-L, Kuo GC, et al. A method to increase tetramer staining efficiency of CD8+ T cells with MHC–peptide complexes: therapeutic applications in monitoring cytotoxic T lymphocyte activity during hepatitis B and C treatment. J Immunol Methods 2004;285:71–87. https://doi.org/10.1016/j.jim.2003.11.005.

[9] Schnuriger A, Dominguez S, Guiguet M, Harfouch S, Samri A, Ouazene Z, et al. Acute hepatitis C in HIV-infected patients: rare spontaneous clearance correlates with weak memory CD4 T-cell responses to hepatitis C virus: AIDS 2009;23:2079–89. https://doi.org/10.1097/QAD.0b013e328330ed24.

[10] Guihot A, Oksenhendler E, Galicier L, Marcelin A-G, Papagno L, Bedin A-S, et al. Multicentric Castleman disease is associated with polyfunctional effector memory HHV-8-specific CD8+ T cells. Blood 2008;111:1387–95. https://doi.org/10.1182/blood-2007-03-080648.
